# Chemical composition and broad-spectrum anthelmintic activity of a cultivar of toothache plant, *Acmella oleracea*, from Mizoram, India

**DOI:** 10.1080/13880209.2020.1760316

**Published:** 2020-05-13

**Authors:** Pawi Bawitlung Lalthanpuii, Kholhring Lalchhandama

**Affiliations:** Department of Life Sciences, Pachhunga University College, Aizawl, Mizoram, India

**Keywords:** Alkylamide, cestode, cuticle, fatty alcohol, nematode, scanning electron microscopy, tegument

## Abstract

**Context:**

A variety of *Acmella oleracea* (L.) R.K. Jansen (Asteraceae) is used by the Mizo people of India and Myanmar for intestinal helminthiasis.

**Objective:**

To perform a chemical analysis of the plant extract using gas chromatography-mass spectrometry (GC-MS) and test the anthelmintic activity on intestinal parasites.

**Materials and methods:**

An extract of the aerial parts was prepared in hexane and analysed using GC-MS. Survival test was performed *in vitro* on the cestode, *Taenia tetragona*, and the nematode, *Ascaridia perspicillum*. Concentrations of 1.25, 2.5, 5, 10 and 20 mg/mL, prepared in phosphate-buffered saline (PBS) with 1% dimethylsulphoxide (DMSO), were tested. Negative control was maintained in PBS with DMSO, and albendazole was used as a reference drug. Each treatment consisted of six worms and was done until death was confirmed. Scanning electron microscopy was used to describe the structural changes.

**Results:**

Nineteen compounds were detected. The major compounds were fatty alcohols such as 3,7,11,15-tetramethylhexadec-2-en-1-ol and (9*Z*)-9-hexadecen-1-ol. Important bioactive compounds including an alkylamide, *N*-isobutyl-(2*E*,4*Z*,8*Z*,10*E*)-dodecatetraenamide, and a triterpenoid, lupeol, were also confirmed. The lethal concentration (LC_50_) of the plant extract was 5128.61 ppm on *T. tetragona* and 8921.50 ppm on *A. perspicillum*. Tegumental shrinkage, erosion of microtriches, and distortion of the suckers were observed on the cestode. The nematode showed collapse of the lips and shrunk cuticle.

**Conclusions:**

*Acmella oleracea* contains important bioactive compounds, which are responsible for the broad-spectrum anthelmintic activity. Further study on the pharmacology of the compounds is warranted.

## Introduction

Helminthiases are among the neglected tropical diseases that attract less attention than other infections chiefly because their immediate symptoms are not life-threatening. They are the major cause of morbidity in humans and decreased productivity in veterinary livestock. In fact, they are now the most prevalent infectious diseases in humans. The World Health Organization (WHO) reports in 2019 that soil-transmitted helminths infect 1.5 billion people (WHO 2019a), while schistosomiases alone accounts for 220 million cases (WHO 2019b), thereby surpassing malaria (at 219 million cases) as the most prevalent infection. It is also vitally crucial to admit that the situation due to helminthiasis is exacerbated by a pervasive anthelmintic resistance in major helminths to all available anthelmintic drugs. The need for improvements of these drugs and alternative sources of drugs is utterly compelling (Becker et al. 2018; Schulz et al. 2018).

Among well-established medicinal plants, *Acmella oleracea* (L.) R.K. Jansen (Asteraceae) is interesting because of its multifaceted applications in traditional medicines and cuisines (Abeysiri et al. 2013). The common name, toothache plant, is given owing to its practical usage in dental health care. It is commonly consumed either cooked or raw as a vegetable or used as food seasoning because of its unique menthol-like minty flavour (Paulraj et al. 2013). It is used in the treatment of anaemia, cancer, constipation, diuresis, fever, flatulence, inflammation, liver abscess, peptic ulcer, and ulcer (Dubey et al. 2013). It is also used in severe malaria cases and has been shown to be effective on malarial parasites (Spelman et al. 2011). In addition, it is known in Indian medicine as an aphrodisiac and is used as a therapy for impotency. It is also used for treating articular rheumatism, dysentery, snakebite, and tuberculosis (Prachayasittikul et al. 2013). The plant extract has been successfully tested for anti-inflammatory (Kim et al. 2018), analgesic, antipyretic (Chakraborty et al. 2010), antimicrobial, antioxidant (Savadi et al. 2010), and insecticidal activities (Simas et al. 2013).

The Mizo people of India and Myanmar have cultivated this plant as a common vegetable and have produced a variety. This variety is easily noticeable with its highly serrated and corrugated leaves, and the dome-shaped and completely yellow inflorescence, which are not found in the type species. In their traditional medicine, the plant is a versatile remedy for gastric problems, headaches, dysentery, oral and dental infections, rheumatism and stuttering in children. Its pungent odour is used to ward off mosquitos and other insects (Lalthanpuii et al. 2017). One of its most unique applications among the Mizo people is as an anthelmintic agent in non-specific intestinal helminthiases. Therefore, it is pertinent to investigate the medicinal property of this plant for its bioactive compounds and actual anthelmintic activity.

## Materials and methods

### Preparation of plant material

*Acmella oleracea* was harvested in November 2018 from a plantation in Ngopa, a village in Mizoram, India, located between 23.8861°N and 93.2119°E. The specimen was authenticated by Ashiho Asosii Mao, botanist (Scientist F) at the Botanical Survey of India, Shillong, and catalogued (PUC-A-17-1) in the herbarium section of Pachhunga University College, Aizawl, India. The aerial parts were dried in the shade at room temperature. The hexane extract was prepared in a 5 L Soxhlet apparatus. The extract slurry was concentrated by evaporating and recovering the solvent in a vacuum rotary evaporator (Buchi Rotavapor^®^ R-215). The semi-solid extract obtained was stored at 4 °C until further use.

### Gas chromatography-mass spectrometry

The plant extract was analysed in a single quadrupole GC-MS system (Thermo Scientific TRACE™ 1300 ISQ™ LT). Solution of the extract was prepared in acetonitrile (50 mg in 3 mL). As a stationary phase, a non-polar column TR-5MS (260F142P, 30 m × 0.25 mm × 0.25 µm with film thickness of 0.25 μm) was used. The injector port and oven temperature were set 250 °C. Helium was used as a carrier gas, and was released into the oven chamber at a constant flow rate of 1 mL/min. Sample was injected in a volume of 1 μL in split mode at the splitting ratio 1:50. The mass spectrometer was run with an ionization electron energy of 70 eV. Ion source and transfer line temperature were set at 250 °C. The total running duration was 55 min. The final chromatogram was generated with Thermo Scientific™ Xcalibur™ software. Compounds were identified based on their retention time, chemical formula and molecular weight from libraries of Wiley Registry™ and National Institute of Standards and Technology database.

### Anthelmintic efficacy

*In vitro* anthelmintic activity was studied on the cestode, *Taenia tetragona*, and the nematode, *Ascaridia perspicillum.* The study was approved by the Institutional Ethics Committee of Pachhunga University College (PUC-IAEC-2016-Z2 of 10/08/2016). Freshly killed fowls were obtained from the Rural Slaughter House, Aizawl, Government of Mizoram. The helminth parasites were collected from the intestines of six naturally infected local fowls, *Gallus gallus domesticus* Linn. Acmella *oleracea* hexane extract was prepared in different concentrations (*viz.* 1.25, 2.5, 5, 10, and 20 mg/mL) by dissolving in 0.9% neutral phosphate-buffered saline (PBS) with 1% dimethylsulphoxide (DMSO). Albendazole (ZENTEL^®^, a product of GlaxoSmithKline, with a standard dosage of 20 mg/mL) was prepared similarly as a reference drug. Control consisted only of PBS with DMSO. Batches of two worms were introduced into each media maintained at 37 ± 1 °C, and each test was done in triplicates.

Anthelmintic efficacy was assessed in terms of duration of survival. Data were presented as normalized statistical means against control ± standard deviation. The lethal concentration (LC_50_) was calculated using Probit analysis. Significance of the anthelmintic activity was determined using Student’s *t*-test, and the level of significance was considered when *p* value was less than 0.05.

### Scanning electron microscopy

Worms in control media and those treated with 20 mg/mL of the plant extract were processed for scanning electron microscopy. They were fixed in 10% neutral formaldehyde (buffered with 0.1 M sodium cacodylate) at 4 °C for 4 h. Secondary fixation was done with 1% osmium tetroxide at 4 °C for 1 h. Dehydration was done in acetone. After treating with tetramethylsilane for 15 min, they were dried in an air-drying chamber at 25 °C. They were mounted on metal stubs and sputter coated with gold in JFC-1100 (JEOL Ltd., Tokyo, Japan) ion-sputtering chamber. Finally, they were observed under a JSM-6360 scanning electron microscope (JEOL Ltd., Tokyo, Japan) at an electron accelerating voltage of 20 kV.

## Results

### GC-MS analysis

**Figure 1.**
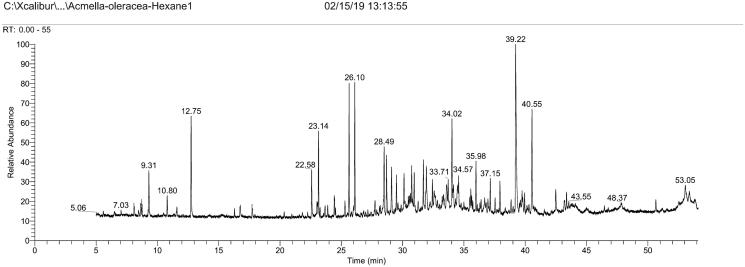
Gas chromatogram of the hexane extract of *A. oleracea.* Total retention time is 55 min.

Gas chromatogram of *A. oleracea* extract is shown in Figure 1, and the list of corresponding chemical compounds identified from mass spectra is given in Table 1. In total, 19 compounds were identified. 3,7,11,15-Tetramethylhexadec-2-en-1-ol was by far the most abundant compound with relative abundance of 99.8%, followed by 9-hexadecen-1-ol (80.4%) and *N*-isobutyl-(2*E*,4*Z*,8*Z*,10*E*)-dodecatetraenamide (67%). Other major compounds were 2,5-dimethyl-5-hexen-3-ol, 6,10,14-trimethylpentadecan-2-one, and propanoic acid. An interesting bioactive compound, lupeol or (3β)-lup-20(29)-en-3-yl acetate was also detected in moderate amount.

**Table 1.** Compounds identified from the hexane extract of *A. oleracea* using GC-MS.

Sl No	Retention time (minute)	Relative abundance (%)	Compound	Formula	Molecular weight (Da)
1.	5.06	14.2	5,9-Dimethyl-1-decanol	C_12_H_26_O	186
2.	7.03	15.4	9-Octadecenamide	C_18_H_35_NO	281
3.	9.31	35.5	2,5-Dimethyl-1,5-heptadiene-3,4-diol	C_9_H_16_O_2_	156
4.	10.80	22.9	(*E*)-4,4-Dimethyl-2-pentene	C_7_H_14_	98
5.	12.75	63.2	2,5-Dimethyl-5-hexen-3-ol	C_8_H_16_O	128
6.	22.58	35.8	Pentanoic acid, isobutyl 3-hydroxy-2,2,4-trimethylpentanoate	C_12_H_24_O_3_	216
7.	23.14	55.5	Propanoic acid, 2-methyl,3-hydroxy-2,2,4-trimethylpentyl ester	C_12_H_24_O_3_	216
8.	26.10	80.4	(9*Z*)-9-Hexadecen-1-ol	C_16_H_32_O	240
9.	28.49	47.9	Caryophyllene oxide	C_15_H_24_O	220
10.	33.71	31.2	7-Hydroxy-6, 9*a*-dimethyl-3-methylene-decahydro-azuleno[4,5-*b*] furan-2,9-dione	C_15_H_20_O_4_	264
11.	34.02	61.7	6,10,14-Trimethylpentadecan-2-one	C_18_H_36_O	268
12.	34.57	33.0	Spiro[tricyclo[4.4.0.0(5,9)]decane-10,2-oxirane] 1-methyl-4-isopropyl-7,8-dihydroxy-, 8(*S*)-	C_15_H_24_O_3_	252
13.	35.98	40.3	8-Acetyl-5,5-dimethyl-nona-2,3,8-trienoic acid, methyl ester	C_14_H_20_O_3_	236
14.	37.15	31.13	Butyl 4,7,10,13,16,19-docosahexaenoate	C_26_H_40_O_2_	384
15.	39.22	99.8	3,7,11,15-Tetramethylhexadec-2-en-1-ol	C_20_H_40_O	296
16.	40.55	67.0	*N*-Isobutyl-(2*E*,4*Z*,8*Z*,10*E*)-dodecatetraenamide	C_16_H_25_NO	247
17.	43.55	20.0	Bufa-20,22-dienolide, 14,15, -epoxy-3,11-dihydroxy-, (3*a*,5*a*,11*a*,15*a*)	C_24_H_32_O_5_	400
18.	48.37	19.4	9-Desoxo-9-x-acetoxy-3,8,12-tri-*O*-acetylingol	C_28_H_40_O_10_	536
19.	53.05	28.3	(3β)-Lup-20(29)-en-3-yl acetate	C_32_H_52_O_2_	468

### Anthelmintic efficacy

The anthelmintic efficacy of albendazole and *A. oleracea* extract on the cestode, *T. tetragona*, is given in Table 2. Significant concentration-dependent effects were seen in all the tests for both the drug and the plant extract. Normalized survival values against control indicated that albendazole took 23.76 ± 1.93 h and 4.39 ± 0.88 h to kill all the worms at the lowest and highest concentrations, respectively. The plant extract took 72.13 ± 0.94 h and 31.65 ± 2.05 h to kill at similar concentrations. Table 3 shows the efficacy of albendazole and *A. oleracea* extract on *A. perspicillum*. Albendazole took 25.17 ± 0.78 h and 1.77 ± 0.44 h to kill all the nematodes at the lowest and highest concentrations respectively. While the plant extract took 89.90 ± 1.06 h and 41.70 ± 0.61 h to kill at similar concentrations. LC_50_ of the plant extract was 5128.61 ppm on *T. tetragona* and 8921.50 ppm on *A. perspicillum*.

**Table 2.** Efficacy of albendazole and the hexane extract of *A. oleracea* on *T. tetragona*.

Treatment	Dose (mg/mL)	Normalized survival time (hour) in mean ± SD	*t* Value	*t* Critical value
Control	0	100.00 ± 2.56	NA	NA
Albendazole	1.25	023.76 ± 1.93*	58.32	2.26
	2.5	020.24 ± 0.58*	74.53	2.45
	5	016.30 ± 0.66*	77.66	2.45
	10	012.15 ± 0.61*	81.85	2.45
	20	004.39 ± 0.88*	86.56	2.45
*A. oleracea* extract	1.25	072.13 ± 0.94*	25.07	2.46
	2.5	062.09 ± 1.35*	32.10	2.31
	5	059.40 ± 1.67*	32.56	2.26
	10	043.99 ± 1.52*	46.15	2.31
	20	031.65 ± 2.05*	51.10	2.23

* Significantly different at *p* < 0.05 against control; NA = not applicable; *n* = 6.

**Table 3.** Efficacy of albendazole and the hexane extract of *A. oleracea* on *A. perspicillum.*

Treatment	Dose (mg/mL)	Normalized survival time (hour) in mean ± SD	*t* Value	*t* Critical value
Control	0	100.00 ± 1.21	NA	NA
Albendazole	1.25	025.17 ± 0.78*	127.60	2.26
	2.5	019.26 ± 0.55*	149.00	2.37
	5	012.76 ± 0.57*	160.20	2.37
	10	007.96 ± 1.21*	132.00	2.23
	20	001.77 ± 0.44*	187.30	2.45
*A. oleracea* extract	1.25	089.90 ± 1.06*	015.40	2.23
	2.5	063.75 ± 0.78*	061.72	2.26
	5	056.79 ± 0.53*	080.36	2.37
	10	046.11 ± 0.76*	092.44	2.31
	20	041.70 ± 0.61*	105.60	2.37

* Significantly different at *p* < 0.05 against control; NA: not applicable; *n* = 6.

### Scanning electron microscopy

**Figure 2.**
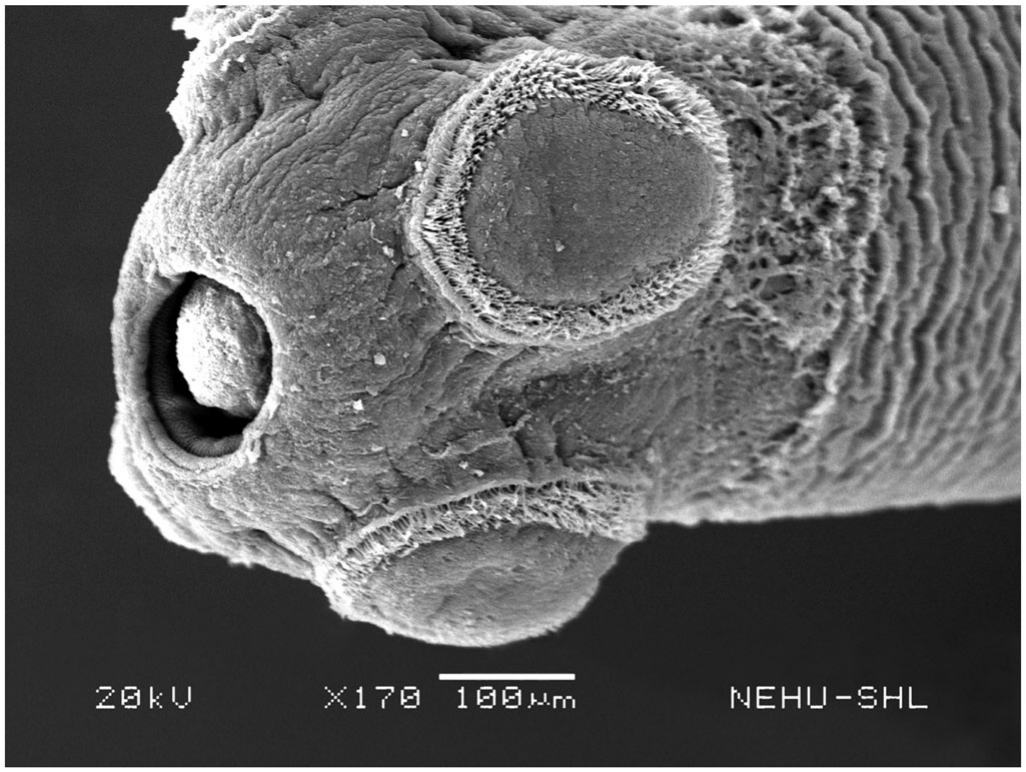
Scanning electron microscopic image of the anterior end of untreated *T. tetragona*. The bulbous scolex and neck are visible. An apical rostellum is surrounded by four oval-shaped suckers.

**Figure 3.**
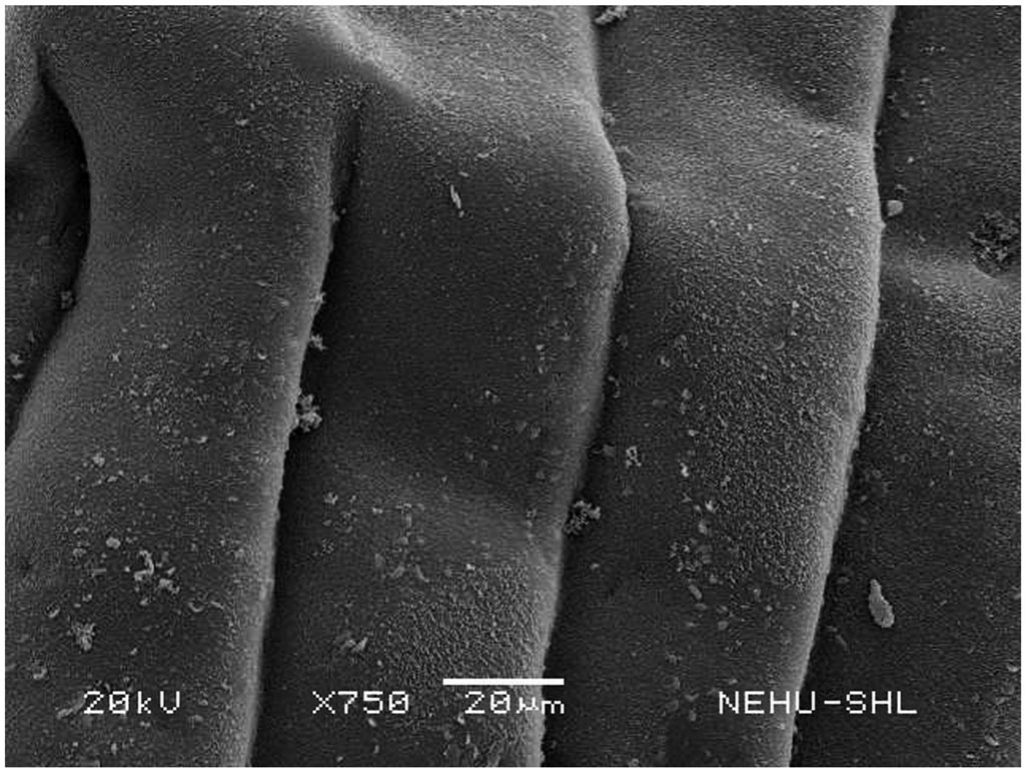
Portion of the body (strobila) of untreated *T. tetragona*. Body segments (proglottids) indicate smooth tegument.

**Figure 4.**
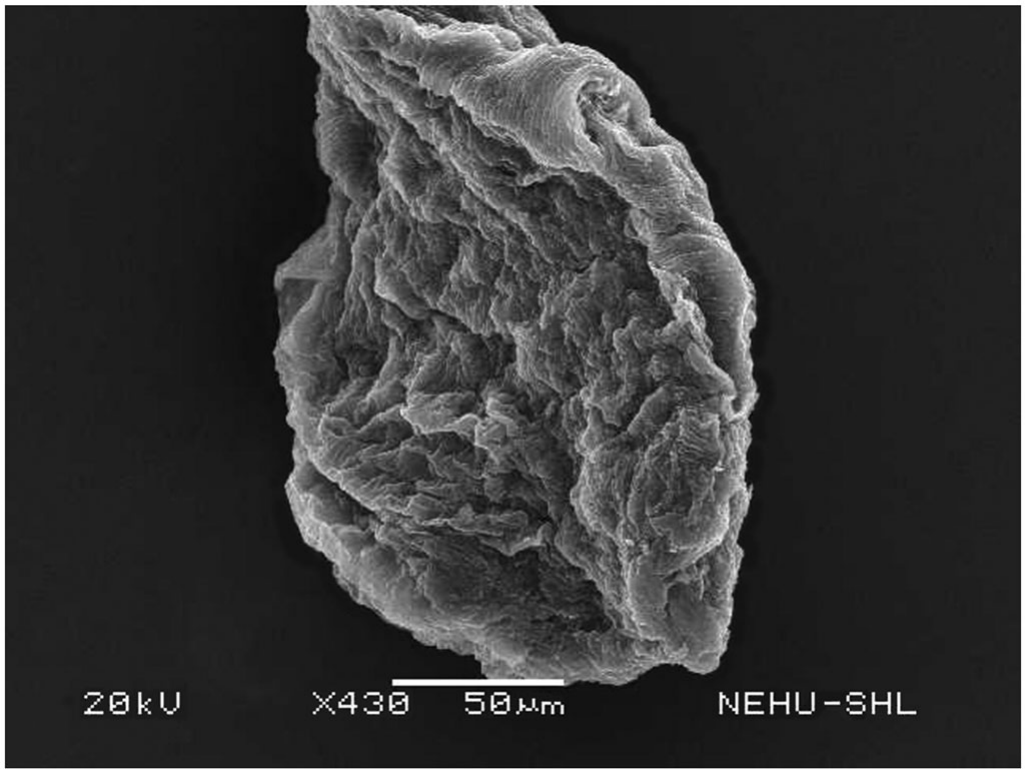
Scanning electron microscopic image of the anterior end of *T. tetragona* treated with *A. oleracea* extract. Due to severe shrinkage suckers are hardly visible on the top and bottom of the scolex. Rostellum is lost.

**Figure 5.**
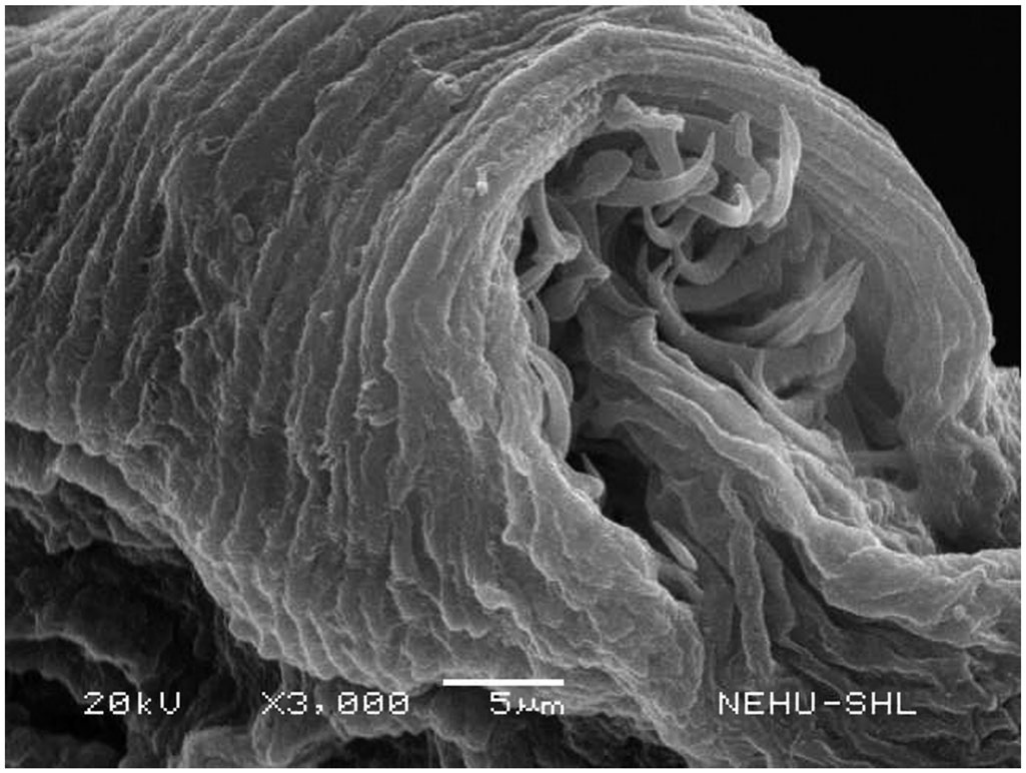
Magnification of the scolex of *T. tetragona* treated with *A. oleracea* extract. A single sucker indicates removal and detachment of spines.

**Figure 6.**
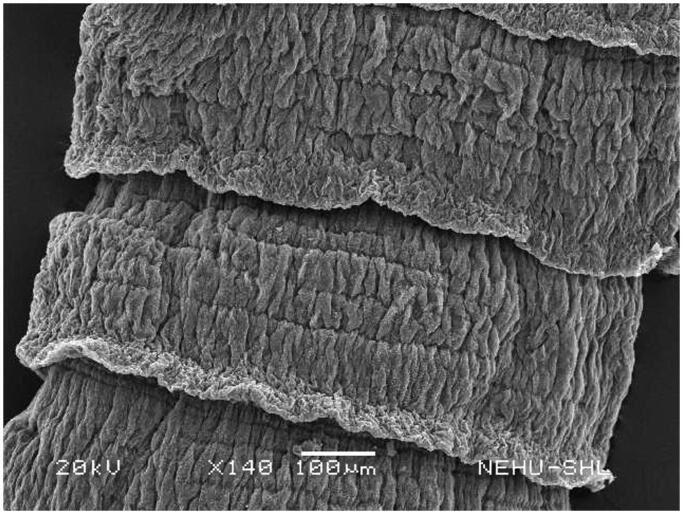
Body segments (proglottids) of *T. tetragona* treated with *A. oleracea* extract. The proglottids are all wrinkled and folded.

Scanning electron microscopy revealed that untreated *T. tetragona* has globular anterior end called sucker, which contains two types of attachment organs, namely rostellum and suckers as shown in Figure 2. The main body (strobila) is composed of a series of body segments called proglottids. The body surface called tegument is smooth because of numerous hair-like microtriches (Figure 3). Remarkable structural changes were seen on *T. tetragona* after treatment with 20 mg/mL of *A. oleracea* hexane extract as shown in Figures 4–6. In Figure 4, the scolex appears as massively distorted. The general body surface called tegument is severely shrunken and wrinkled. A sucker is focussed in Figure 5 showing irregularly oriented spines, the parasitic holdfast devices. Some of the spines are clumped and some are detached. Spines are totally removed at the lower area. The body segments (proglottids) are extensively shrunk and folded. There are no signs of hair-like projections called microtriches indicating complete surface erosion (Figure 6).

**Figure 7.**
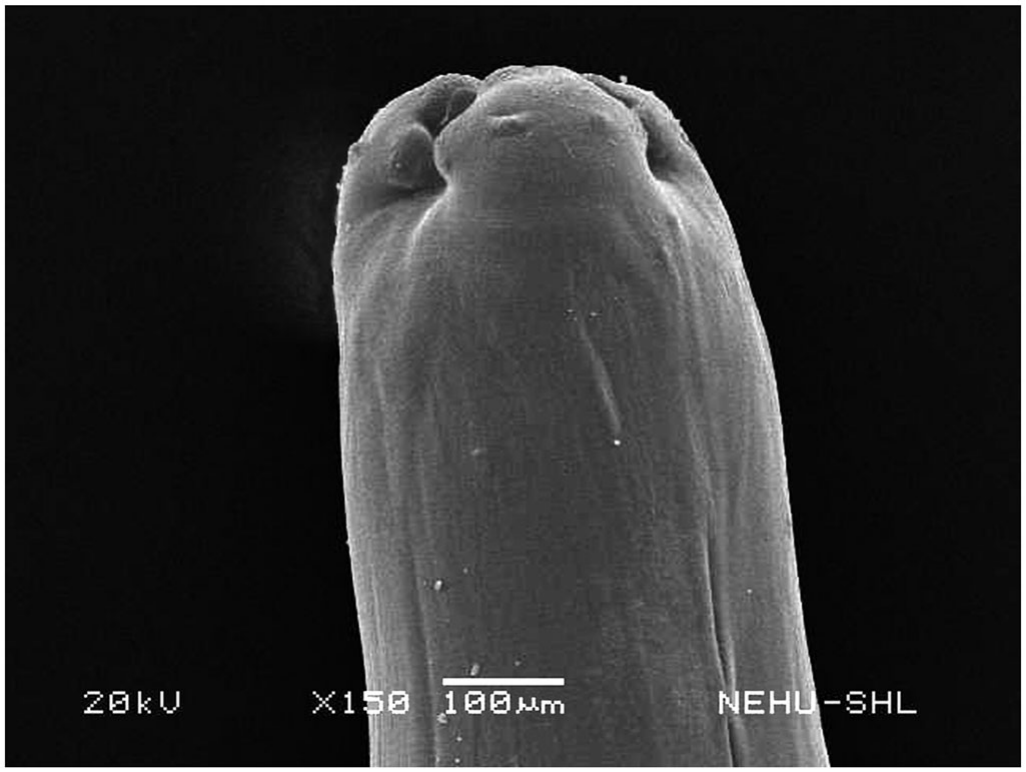
Scanning electron microscopic image of the anterior region of untreated *A. perspicillum*. The three lips are visible. One of the lips has eye-like amphid.

**Figure 8.**
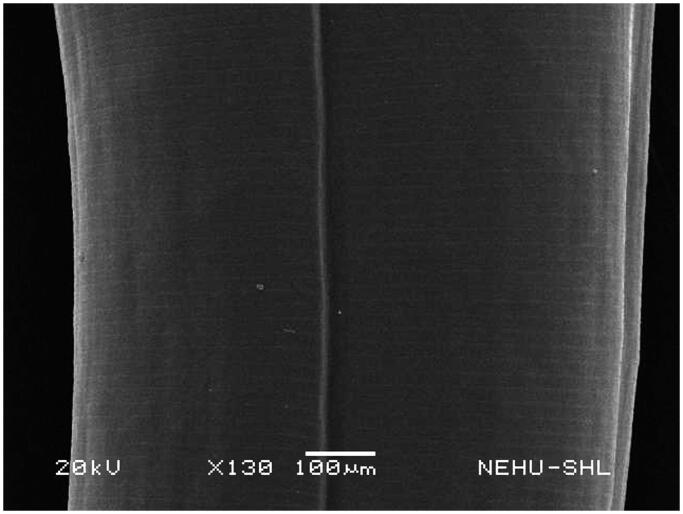
Body surface of untreated *A. perspicillum.* The cuticle is smooth with transverse lines.

**Figure 9.**
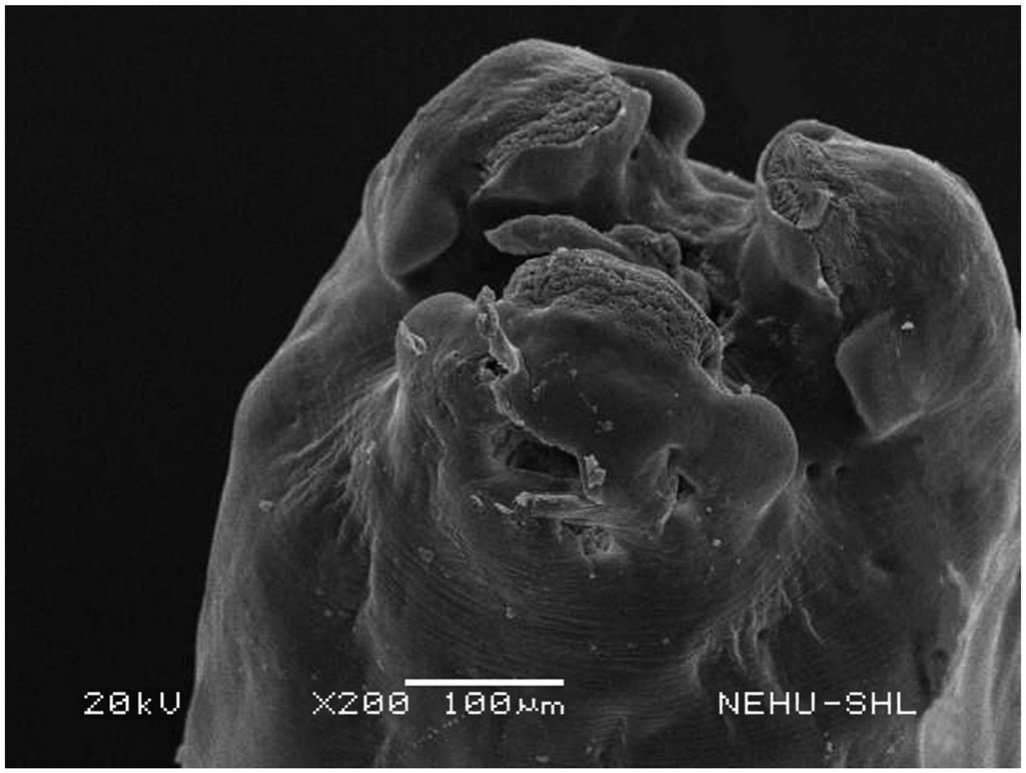
Scanning electron microscopic image of the anterior region of *A. perspicillum* treated with *A. oleracea* extract. The three lips are damaged. There are no signs of eye-like amphids.

**Figure 10.**
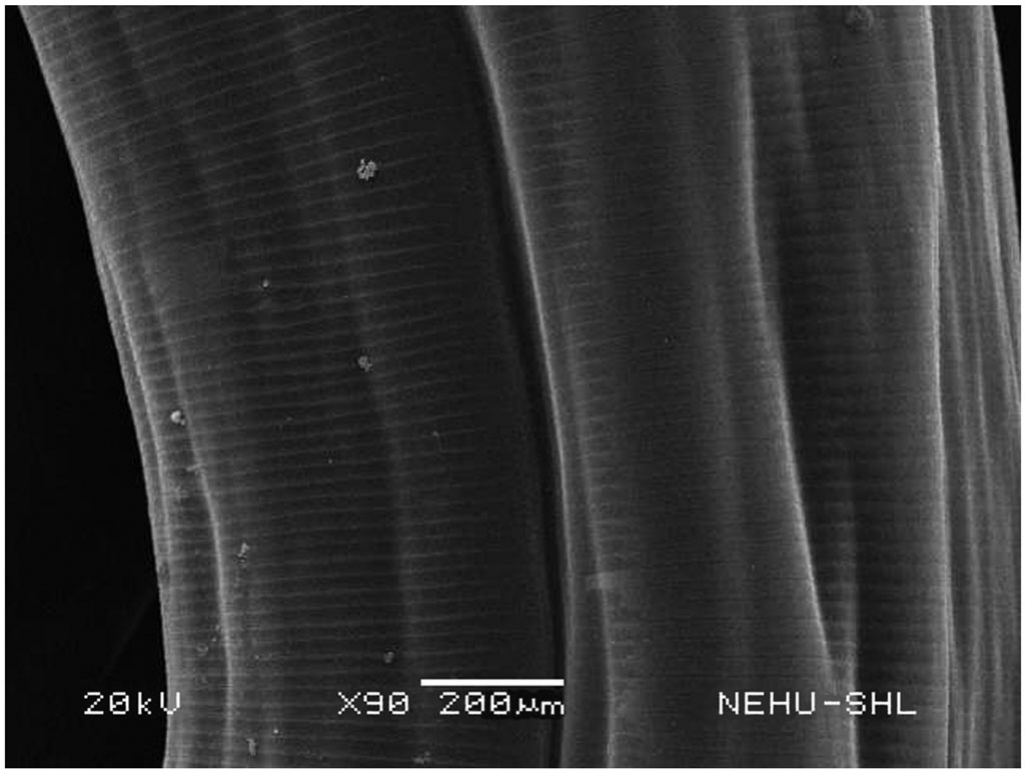
Body surface of *A. perspicillum* treated with *A. oleracea* extract. Shrinkage of the cuticle is evident.

**Figure 11.**
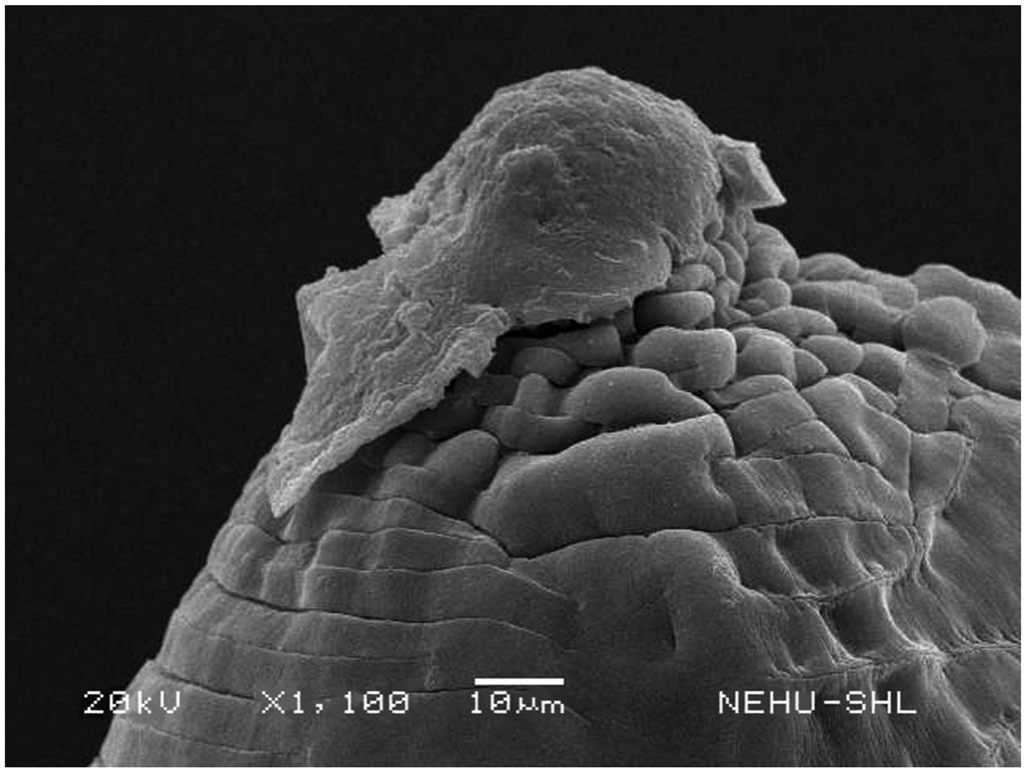
Tail end of *A. perspicillum* treated with *A. oleracea* extract. The cuticle is severely shrunk and folded. The pointed tail is deformed.

Untreated *A. perspicillum* has smooth body surface called cuticle. At the anterior end there are three lips each of which contains eye-like protrusions called amphids (Figure 7). The general body is smooth with transverse striations or rings (Figure 8). The effects of *A. oleracea* hexane extract on *A. perspicillum* are most prominent on the head as shown in Figure 9. The three lips indicate severe damage with a number of scars. The sensory organs, amphids, are lost. There is also shrinkage on the cuticle behind the lips. Shrinkage extends throughout the main body (Figure 10), and is most severe at the posterior region of the body where transverse rings are distorted, and the tail end is abnormally distorted (Figure 11).

## Discussion

*Acmella oleracea* is already known to be a rich source of important bioactive compounds such as amides, α- and β-amyrinester, miricilic alcohol glycosides, sitosterol, saponins, stigmasterol, and triterpenes which are attributed to different biological activities (Lemos et al. 1991; Paulraj et al. 2013). *N*-Isobutyl-(2*E*,4*Z*,8*Z*,10*E*)-dodecatetraenamide recorded here is also reported from other species of *Acmella* (Leng et al. 2011). This compound and other *N-*alkylamides are established to be the principal bioactive compounds in the genus *Acmella*, *Wedelia acapulcensis* var*. parviceps* (S.F.Blake) Strother (Asteraceae), and *Heliopsis longipes* (A.Gray) S.F.Blake (Asteraceae). These compounds have been shown to have analgesic, neuroprotective, antioxidant, antimutagenic, anticancer, anti-inflammatory, antimicrobial, and insecticidal activities (Barbosa et al. 2016). Lupeol is also interesting because it has been experimentally demonstrated for antiparasitic activities in leishmaniasis and trypanosomiasis (Machado et al. 2018), and anticancer (Bhattacharyya et al. 2017), and anti-inflammatory activities (Shen et al. 2019).

Structural damages observed on *T. tetragona* in this study are among the hallmark effects of anthelmintic compounds. The body surfaces of helminths are sensory in function and constitute the direct interface between the parasites and the host. As such, they are the primary target sites of anthelmintics (Taman and Azab 2014). As their parasitic adaptation, cestodes lack internal digestive system so that their body surface (specifically called tegument) with its hair-like microtriches serves as an organ of chemoreception and absorption of nutrients from the host’s intestine (Rana and Misra-Bhattacharya 2013). Thus, anthelmintics primarily attack the tegument.

Albendazole and flubendazole were experimentally shown to cause damage on the rostellum, development of swellings or blebs on the tegument, erosion of the microtriches, and increased vesiculation on the cestode, *Echinococcus granulosus* (Elissondo et al. 2006). Albendazole alone also caused severe shrinkage, obliteration of microtriches and disintegration of the tegument in *Raillietina echinobothrida* (Lalchhandama 2010). A combination therapy of albendazole-praziquantel against *Mesocestoides corti* caused important structural damages including deformity of the suckers, erosion of the tegument and disintegration of the microtriches (Markoski et al. 2006).

Highly lipophilic in nature, albendazole is one of the most diffusible anthelmintic molecules through the cuticle of nematodes. This property is important in drug action because the cuticle is chemically a highly specialized proteinaceous complex and is impermeable to most molecules. It has been shown that the drug enters through the cuticle of *Haemonchus contortus* and *Ascaris suum* by passive diffusion (Alvarez et al. 2007). In the cuticular and the underlying muscle tissues, albendazole prevents polymerization of microtubules by competitively binding to β-tubulins to cause paralysis and death (Abongwa et al. 2017). Ivermectin diffuse through the cuticle and effectively inhibits muscle contraction in *Caenorhaditis elegans*, *H. contortus* and *Oncocerca ochengi* (Yates et al. 2003). Cyclotides, a family of plant peptides, are known to directly attack the cuticle of *H. contortus* and *Trichostrongylus colubriformis* (Colgrave et al. 2010). Natural cysteine proteinases are also found to cause disintegration of the cuticular proteins in different nematodes (Luoga et al. 2015). Therefore, our observations on the structural changes on the cuticle of *A. perspicillum* indicate that *A. oleracea* also directly targets the cuticle to cause deleterious anthelmintic effects.

## Conclusions

*Acmella oleracea* as an anthelmintic plant in Mizo traditional medicine is effective on both the cestode, *T. tetragona* and the nematode, *A. perspicillum*. The plant extract caused structural damages on the tegument of cestodes and cuticle of nematodes, thereby exerting characteristic broad-spectrum anthelmintic effects. Detection of alkylamide and lupeol from the plant extract is interesting as these compounds are established for their specific pharmacological activities. These compounds are among the lead molecules with anthelmintic properties and this necessitates further investigation to understand the specific compounds and their precise biological actions.
